# An Evolutionary Concept Analysis of Spiritual Competence in Nursing

**DOI:** 10.1111/jan.16660

**Published:** 2024-12-01

**Authors:** Nadeen Sami Alshakhshir, Anne L. Ersig, Earlise Ward, Verna L. Hendricks‐Ferguson, Kathleen E. Montgomery

**Affiliations:** ^1^ School of Nursing University of Minnesota Minneapolis Minnesota USA; ^2^ School of Nursing University of Wisconsin–Madison Madison Wisconsin USA; ^3^ School of Medicine and Public Health University of Wisconsin–Madison Madison Wisconsin USA; ^4^ School of Nursing Saint Louis University Saint Louis Missouri USA

**Keywords:** competence, nurse, nursing, spiritual care, spiritual competence, spirituality

## Abstract

**Aims:**

Clarify the concept of spiritual competence in the context of nursing through evidence‐based extraction of attributes, antecedents and consequences.

**Design:**

Concept analysis.

**Methods:**

Rodgers' evolutionary method of concept analysis guided this analysis, and we followed the Preferred Reporting Items for Systematic Reviews and Meta‐Analyses extension for Scoping Reviews.

**Results:**

An analysis of 45 studies clarified the concept of spiritual competence in the context of nursing. Spiritual competence in nursing is a dynamic process that, once initiated by nurses' spiritual awareness, promotes their spiritual well‐being, their spiritual knowledge and skills and their sense of spiritual‐care self‐efficacy.

**Conclusion:**

A comprehensive understanding and clear conceptual definition of the concept of spiritual competence in nursing will enable researchers to overcome barriers and foster nurses' development of spiritual competence to provide spiritual care in nursing.

**Implications for the Profession and Patient Care:**

The clarified concept of spiritual competence in nursing can help to shape education and clinical‐training programs for nurses in a way that supports their ability to provide spiritual care and promote patient well‐being and positive health outcomes.

**Patient or Public Contribution:**

No patient or public contribution was essential to the conduct of this research.

**Summary**

- What does this paper contribute to the wider global clinical community?
  ○ Clarification of the concept of spiritual competence in nursing to guide future research, education and clinical practice.
  ○ Acknowledgment of the essential role of spiritual competence in nurses' ability to promote patient well‐being.

## 1 Background

Spirituality is a complex construct that is related to all dimensions of being. It is defined as “a dynamic and intrinsic aspect of humanity through which persons seek ultimate meaning, purpose, and transcendence and experience relationship to self, family, others, community, society, nature, and the significant or sacred’’ (Puchalski et al. 2009, p.11). The construct of spirituality has been recognised as an essential aspect of human health and illness for decades. Florence Nightingale, the founder of modern nursing, described how spirituality in ill patients led to positive patient outcomes ranging from physical healing to states of transcendence (Burkhardt 1998).

Several professional nursing organisations have shared tenets about the importance of integrating spirituality into whole‐person care. The American Holistic Nurses Association (2016) have proposed that spiritual integration should be an essential part of holistic person‐centred care. The Hospice and Palliative Nurses Association (2022) provides guidelines for the integration of spiritual care into nursing practices devoted specifically to end‐of‐life contexts. A tenet of the American Association of Colleges of Nursing (2021) is that spiritual integration is an essential competency that should be cultivated in nursing education.

Spirituality is associated with positive health outcomes. For example, it has been shown to improve people's mental health and overall quality of life while specifically reducing people's mortality risk, depressive symptoms, suicidal behaviours and engagement in risky behaviours (e.g., smoking and substance use; Balboni et al. 2022; Puchalski et al. 2009). These positive outcomes have been observed among different age groups across the lifespan (Balboni et al. 2022). Because nurses are responsible for promoting the health and well‐being of their patients, and because spirituality is key to this task, nurses must learn how to show spiritual support to patients, particularly in times of need and distress. In ascertaining how nurses fulfil their spiritual role, it is critical to first assess their ability and willingness to perform the role.

Spiritual care is also recognised as being essential to holistic nursing care (Ramezani et al. 2013). Spiritual care must not only relieve spiritual distress in patients but also enable them to self‐heal through spiritual means (Puchalski et al. 2009). Ramezani et al. (2013) identified the following seven characteristics that spiritually competent nurses possess: (1) a healing presence, (2) a therapeutic use of the self, (3) intuitive sense, (4) an ability to explore spiritual perspectives, (5) patient‐centredness, (6) an ability to perform meaning‐centred therapeutic intervention and (7) an ability to foster a spiritually nurturing environment. Nurses can be spiritually present for patients in times of illness, loss and other forms of suffering (Baldacchino 2006; Ramezani et al. 2013). Spiritual care in nursing contexts has been found to be reciprocal in the sense that both the nurse and the patient can benefit from it (Bangcola 2022). For example, in one study, patients who received spiritual care from nurses reported experiencing improvement in their spiritual well‐being, spiritual healing, psychological adaptation and overall satisfaction and the nurses shared perceptions of experiencing increased spiritual awareness and job satisfaction (Ramezani et al. 2013). Despite the vast literature detailing the positive outcomes of spirituality and spiritual care for both nurses and patients, there are obstacles that may hinder nurses' realisation of the aforementioned positive outcomes. Some of these obstacles include heavy workloads, limited knowledge of spirituality and feelings of incompetence with respect to the provision of spiritual care (Babamohamadi et al. 2022; Baldacchino 2006; Özakar Akça, Gülnar, and Özveren 2022).

Competence also applies to other aspects of nursing such as physical assessment and medication administration. Competent nurses are those who (1) possess sufficient knowledge; (2) have mastered specific patient centred focused skills; (3) use professional judgement; (4) respect and adhere to professional standards; (5) maintain positive interpersonal relationships; (6) use knowledge and skills in situationally appropriate ways and (7) evaluate success according to rigorous outcome measurements (Church 2016). These characteristics of competence in nursing rely heavily on nurses' therapeutic use of knowledge, skills and the self. Because competence in nursing involves the application of particular practices tailored to particular contexts, nurses must learn how to apply spiritual practices to contexts requiring some form of spiritual care (Church 2016; Damsma‐Bakker and van Leeuwen 2021; Ramezani et al. 2013). Put simply, to provide competent spiritual care, nurses must be spiritually competent (Southard et al. 2020; Ramezani et al. 2013). Despite widespread use of the term in the literature, a clear definition or conceptual analysis of spiritual competence in nursing does not exist. Researchers have created three conceptual models of nurse‐based competence in ‘spiritual care’ as a way to improve the education and clinical training of nurses with respect to spiritual care (Attard, Ross, and Weeks 2019; Campinha‐Bacote 1995; Cao et al. 2020). Although these conceptual models strengthen our understanding of *spiritual‐care competence*, they do not explicitly address *spiritual competence*.

Many limitations in providing spiritual care to patients have been identified in the literature. One of the most important is nurses' hesitancy to provide spiritual care to patients (Babamohamadi et al. 2022; Baldacchino 2006). Our understanding of the influence of ability and willingness on nurses' provision of spiritual care is limited. It stands to reason, however, that for nurses to be able and willing to provide good spiritual care, they must possess a reasonable degree of spiritual competence. Because there is still a lack of clarity regarding the possible factors influencing nurses' ability and willingness to provide spiritual care, more research is needed to clarify the concept of spiritual competence in nursing.

### 1.1 Aim

We aimed to perform a concept analysis that clarifies the concept of spiritual competence in nursing through evidence‐based identification of attributes, antecedents and consequences and that thus improves the education and clinical training of nurses with respect to spiritual care.

## 2 Methods

Rodgers' (1989) evolutionary concept analysis method guided this concept analysis. Concept analyses aim to clarify the definition of concepts in specific contexts. Rodgers' evolutionary method has three iterative phases. In the initial phase, the researcher identifies three critical factors: the concept selected for analysis, the context of the concept and the literature supportive of the proposed concept analysis. In the second phase, in which core analysis takes place, the researcher identifies three further factors: the concept's related and surrogate terms; the concept's attributes, antecedents and consequences and examples of the concept's use. In the third and final phase, known as the ‘further analysis’ phase, the researcher identifies implications that the findings from the second stage may have for further development of the concept in research (Rodgers, 1989, as cited in Tofthagen and Fagerstrøm (2010)). For reporting the findings, we followed The Preferred Reporting Items for Systematic Reviews and Meta‐Analyses extension for Scoping Reviews (PRISMA‐ScR) checklist (Tricco et al. 2018).

### 2.1 Data Sources

A search strategy was developed by the co‐authors in conjunction with an experienced medical librarian. The following online databases were searched: CINAHL, PsycINFO, PubMed, Academic Search Premier, Scopus and SocINDEX databases for the following terms: *spiritual* (OR) *religion* (OR) *faith* (OR) *belief system* (OR) *religious beliefs* (AND) *nurse* (OR) *physician* (OR) *clinician* (OR) *doctor* (OR) *social worker* (OR) *healthcare worker* (AND) *knowledge* (OR) *attitude* (OR) *competency* (OR) *belief* (OR) *perception* (OR) *awareness*. All database searches were limited to English‐language articles. There was no limit on publication dates for this search. We followed three inclusion criteria: (1) the study populations needed to include nurses or nursing students, (2) the study itself needed to focus on spiritual competence and (3) the study needed to include original empirical research.

In selecting and reviewing the articles identified through our literature search, we relied on Covidence systematic review software (Veritas Health Innovation 2023) to (1) screen titles and abstracts in order to select those articles most relevant to the concept of interest, (2) perform full‐text reviews of the relevant articles to ensure that the final set of articles truly focused on the concept and (3) extract data from the final set of included articles.

**FIGURE 1:**
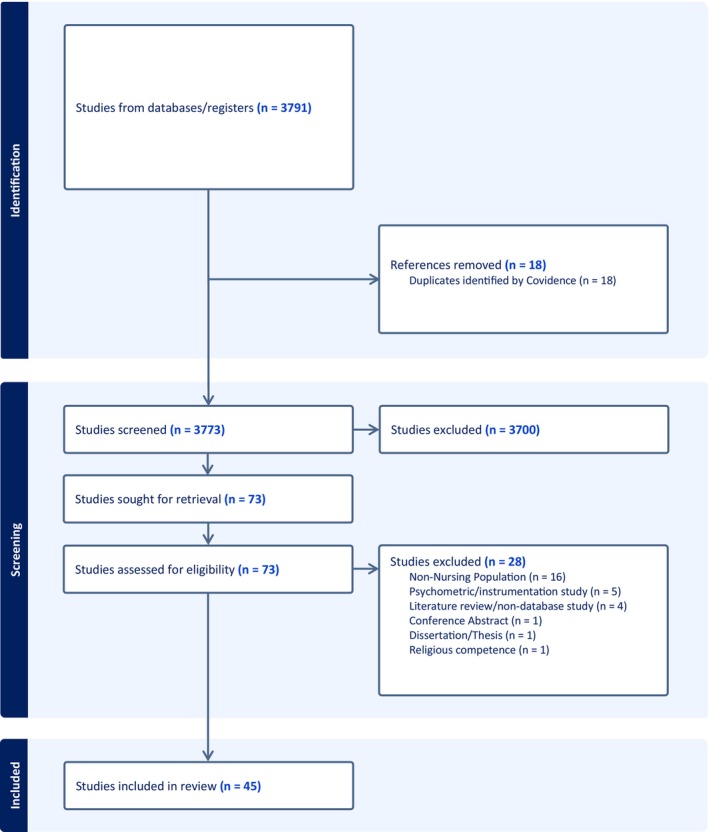
PRISMA flow diagram of the data‐collection process.

The results of the literature search are presented in Figure 1 using a Preferred Reporting Items for Systematic Reviews and Meta‐Analyses (PRISMA) diagram. The initial search returned 3791 articles, of which 18 were duplicates. We reviewed the titles and abstracts of 3773 articles to determine their relevance to the concept of spiritual competence in nursing. We eliminated 3700 articles that failed to reference the concept or population and context of interest. We reviewed the full text of the remaining 73 articles: 45 of them, published between 2006 and 2023, met the inclusion criteria and were included in the analysis.

### 2.2 Data Extraction and Analysis

**FIGURE 2:**
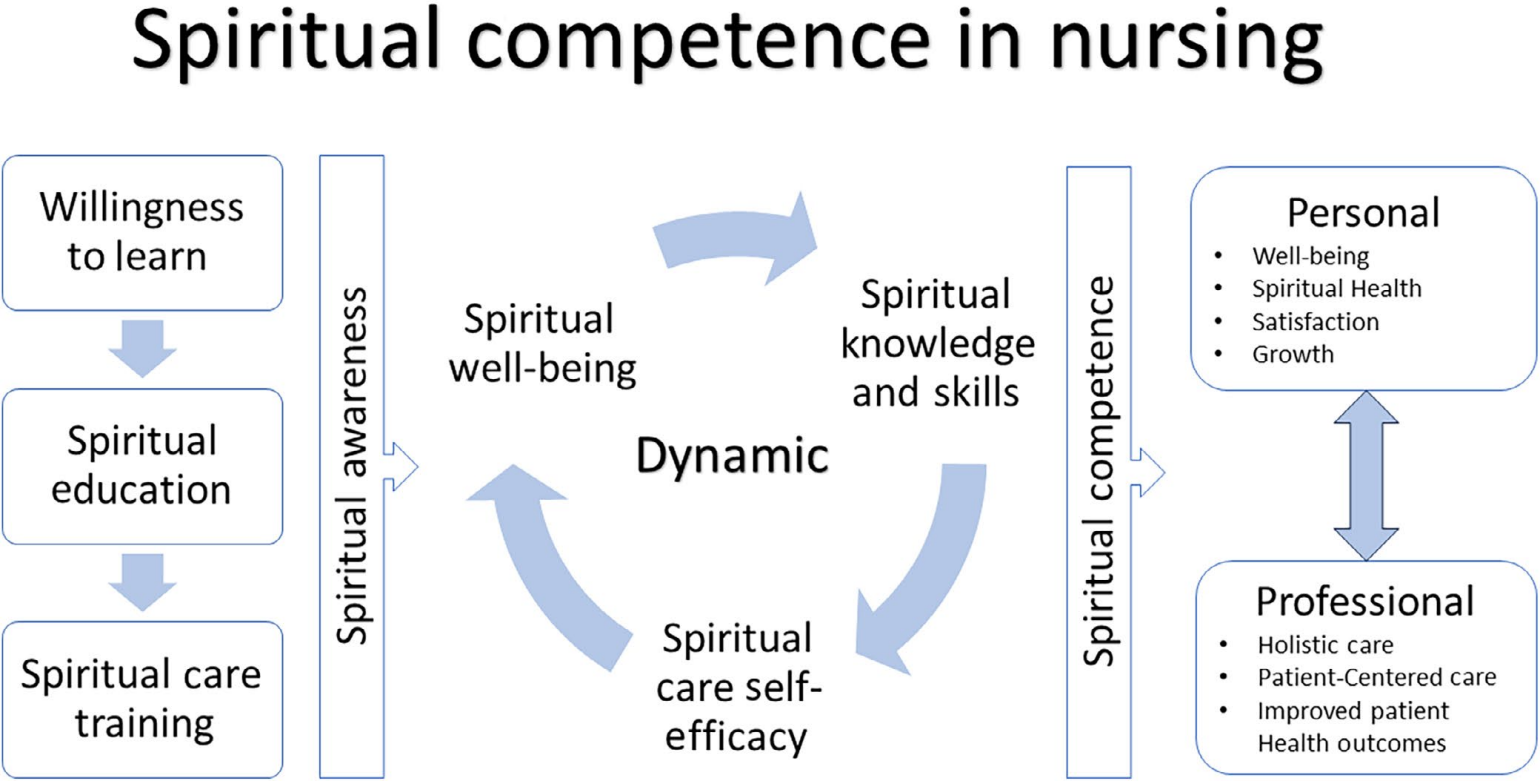
Attributes, antecedents and consequences of spiritual competence in nursing.

The final sample of 45 articles consisted of 40 quantitative, 2 qualitative and 3 mixed‐methods studies. Most of the quantitative studies were descriptive cross‐sectional studies. All studies were in the nursing discipline and had been conducted in academic or clinical settings. Each article's data were extracted in Covidence and exported to an Excel data sheet for data management. Extracted data included surrogate and related terms, the definition of ‘spiritual competence’ (if provided), study purpose, a summary of the methods section and the attributes, antecedents and consequences of spiritual competence identified in the article. We closely examined the identified attributes, antecedents and consequences of spiritual competence in nursing to determine which were essential. Figure 2 presents the identified attributes, antecedents and consequences of spiritual competence from the relevant articles.

## 3 Results

### 3.1 Surrogate and Related Terms

Surrogate terms are technical synonyms: words or phrases that have the same meaning as a selected word or phrase (Tofthagen and Fagerstrøm 2010). In the present study, our selected term is ‘spiritual competence’. Multiple articles use ‘spiritual‐care competence’ as a surrogate term for ‘spiritual competence’. However, in meaning, the two terms differ from each other. The term ‘spiritual‐care competence’ refers to nurses' ability to provide spiritual care (van Leeuwen and Cusveller 2004; van Leeuwen et al. 2009). To operationalise the concept of spiritual‐care competence, researchers in nursing have used the Spiritual Care Competence Scale (SCCS) (van Leeuwen et al. 2009), which is based on a competency profile that can guide the design of education and training programs targeting spiritual care in the nursing profession (van Leeuwen and Cusveller 2004). The SCCS assesses six competencies: (1) assessment and implementation of spiritual care; (2) professionalisation and improvement of the quality of spiritual care; (3) personal support and patient counselling; (4) referral to spiritual professionals; (5) attitudes toward patient spirituality and (6) communication (van Leeuwen et al. 2009).

Because the concept of spiritual‐care competence focuses on care, it does not typically emphasise spiritual competence. This distinction is subtle yet critical, as nurses can be somewhat competent providers of spiritual care even though they possess only some degree of spiritual knowledge. In short, the concept of spiritual competence is broader in scope than the concept of spiritual‐care competence.

Related terms refer to concepts that are associated with—rather than synonymous or nearly synonymous with—the selected concept (Tofthagen and Fagerstrøm 2010). Terms related to spiritual competence in the field of nursing include “spirituality” (Alshehry 2018; Kalkim, Sagkal Midilli, and Daghan 2018); “cultural competence” (Abu‐Snieneh and Abdelaziz 2022; Bangcola 2022); “ethical competence” (Baldacchino 2006; Ebrahimi et al. 2017); “spiritual intelligence” (Ahmadi et al. 2021) and “attitudes toward death” (Li et al. 2021). Though they are important to know, these and other related terms are not critical to the specific focus of the present study.

### 3.2 Definitions

Among the final 45 articles included in this concept analysis, 13 defined spiritual competence or spiritual‐care competence in nursing. We extracted these definitions verbatim from each article to preserve their meaning (see Table 1). Similarities in definitions included that nurses' spiritual competence (or nurses' spiritual‐care competence) requires knowledge of spirituality and an ability to provide spiritual care (Ahmadi et al. 2021; Alshehry 2018; Babamohamadi et al. 2022; Chen et al. 2020; Ebrahimi et al. 2017; Heidari, Afzoon, and Heidari 2022; Hsieh et al. 2020; Kang et al. 2021; Li et al. 2021; Özakar Akça, Gülnar, and Özveren 2022; Ross et al. 2016; Southard et al. 2020; Wang et al. 2022).

**TABLE 1:** Definitions of ‘spiritual care competence’ and ‘spiritual competence’ in nursing.

Article	Title	Definition
Ahmadi et al. (2021)	‘Perceived Professional Competence in Spiritual Care and Predictive Role of Spiritual Intelligence in Iranian Nursing Students’	‘Professional competence in spiritual care is recognised as a continuously active process defined by three related elements of awareness of human values, empathy with the client and the ability to perform appropriate individual interventions for each client. It also includes seven general competencies: integrating one's individuality and nursing role as a profession; helping the patient to find the meaning of the disease and accepting it; maintaining a positive relationship with patients and their family; relationship with patients, interdisciplinary teams and clinical/educational organisations; providing spiritual care during the four stages of the nursing process (review, planning, implementation and evaluation); observance of ethical issues in care such as confidentiality and protection of information; and providing holistic care’. (p. 2)
Alshehry (2018)	‘Spirituality and Spiritual Care Competence among Expatriate Nurses Working in Saudi Arabia’	‘Spiritual care competencies in nursing involve a set of skills used in professional nursing practice and within the framework of the nursing process, resulting in a positive outcome’. (p. 2)
Babamohamadi et al. (2022)	‘Nursing Students' Professional Competence in Providing Spiritual Care in Iran’	‘Competence in providing spiritual care refers to a collection of skills used in the practice of clinical nursing, such as nurse–patient therapeutic communication, accessibility to patients, active listening, giving value to life and fostering hope by expressing empathy and sympathy, facilitating religious practices for patients with specific religious beliefs, helping patients access a relaxed atmosphere and their religious atmosphere of choice, and helping patients complete any unfinished work and consult with clerics or other religious and spiritual professionals’. (pp. 1832–1833)
Chen et al. (2020)	‘Factors Influencing the Self‐Perceived Competencies in Spiritual Care of Nurses in the Long‐Term Care Facilities’	‘Competency in spiritual care refers to nurses' ability to assess and provide interventions to meet the complicated spiritual needs of patients in collaboration with the multidisciplinary health care team’. (p. 1287)
Ebrahimi et al. (2017)	‘Health Care Providers' Perception of Their Competence in Providing Spiritual Care for Patients’	‘Spiritual competence in spiritual care refers to a set of skills which are used in the clinical nursing processes’. (p. 58)
Heidari, Afzoon, and Heidari (2022)	‘The Correlation between Spiritual Care Competence and Spiritual Health among Iranian Nurses’	‘Spiritual care competence is an active and ongoing process, consisting of increasing awareness of the patient's values, empathetic understanding of their worldviews, and capability for individualized interventions for each patient. Spiritual care competence consists of a set of knowledge, skills, and attitudes that enable nurses to practice holistic care in their caring behavior, while nurses' caring behavior is closely related to their spiritual health’. (p. 2)
Hsieh et al. (2020)	‘Factors Associated with Spiritual Care Competencies in Taiwan's Clinical Nurses: A Descriptive Correlational Study’	‘Spiritual care competency is defined as an ongoing process with three related elements: containing a growing awareness of personal value, developing empathic understanding of clients' worldview and performing adequate individual management for each client’. (p. 1601)
Kang et al. (2021)	‘Hospice Palliative Care Nurses' Perceptions of Spiritual Care and Their Spiritual Care Competence: A Mixed‐Methods Study’	‘Spiritual care competence (SCC) is an evaluation of a nurse's knowledge, skills and attitudes to be able to provide adequate spiritual nursing care to meet a patient's religious and existential needs, including their experiences and questions about the meaning and purpose of life’. (p. 962)
Li et al. (2021)	‘Association between Attitude towards Death and Spiritual Care Competence of Chinese Oncology Nurses: A Cross‐Sectional Study’	‘Spiritual care competence (SCC) refers to an ability to assess and identify patient spiritual needs and implement appropriate interventions to promote patient spiritual health. SCC requires knowledge, attitudes, and skills of spiritual care’. (p. 1)
Özakar Akça, Gülnar, and Özveren (2022)	‘Spiritual Care Competence of Nurses’	‘Spiritual care competence is related to the knowledge skills and attitudes that healthcare professionals need to provide effective spiritual care’. (pp. 225–226)
Ross et al. (2016)	‘Factors Contributing to Student Nurses'/Midwives' Perceived Competency in Spiritual Care’	‘Spiritual care competency has been defined as the knowledge, skills and attitudes needed for delivery of spiritual care’. (p. 446)
Southard et al. (2020)	‘Perceptions of Spiritual Care Education, Competence, and Barriers in Providing Spiritual Care Among Registered Nurses’	‘Spiritual care competence is the ability of the nurse to assess for and provide interventions to care for a patient's spiritual needs. It is defined by the knowledge, skills, and attitudes needed for delivery of spiritual care’. (p. 42)
Wang et al. (2022)	‘Spiritual Care Perceptions, Spiritual Health and Their Relationships with Spiritual Care Competence among Clinical Nurses in China: A Cross‐Sectional Quantitative study’	‘Spiritual care competence refers to the knowledge, attitude and skills of spiritual care possessed by nurses, which can significantly improve the physical and mental health of patients and their satisfaction with the quality of clinical nursing practice services’. (p. 244)

### 3.3 Attributes

Attributes are essential characteristics that define a concept (Tofthagen and Fagerstrøm 2010). We identified the following five attributes of spiritual competence in nursing: (1) spiritual awareness (Adib‐Hajbaghery, Zehtabchi, and Fini 2017; Ahmadi et al. 2021; Baldacchino 2006; Damsma‐Bakker and van Leeuwen 2021; Guo et al. 2023; Irmak and Sagkal Midilli 2021; Kalkim, Sagkal Midilli, and Daghan 2018; Ross et al. 2022, 2016; Semerci et al. 2021; Sezer and Ozturk Eyimaya 2022; van der Vis‐Sietsma, de Man‐van Ginkel, and van Leeuwen 2019; Zhang et al. 2023); (2) spiritual well‐being (Azarsa et al. 2015; Bangcola 2022; Jafari and Fallahi‐Khoshknab 2021; Özakar Akça, Gülnar, and Özveren 2022; Ross et al. 2016, 2018, 2014); (3) spiritual knowledge and skills (Babamohamadi et al. 2022; Connors, Good, and Gollery 2017; Ebrahimi et al. 2017; Guo et al. 2023; Heidari, Afzoon, and Heidari 2022; Hellman, Williams, and Hurley 2015; Irmak and Sagkal Midilli 2021; Kalkim, Sagkal Midilli, and Daghan 2018; Kang et al. 2021; Karaman and Sagkal Midilli 2022; Özakar Akça, Gülnar, and Özveren 2022; Petersen et al. 2017; Petersen and Schiltz 2020; Wang et al. 2022; Zhang et al. 2023); (4) spiritual‐care self‐efficacy (Azarsa et al. 2015; Cheng et al. 2021; Guo et al. 2021; Heidari, Afzoon, and Heidari 2022; Kalkim, Sagkal Midilli, and Daghan 2018; Petersen et al. 2017; Sezer and Ozturk Eyimaya 2022; Shamsi, Khoshnood, and Farokhzadian 2022; Southard et al. 2020; Wang et al. 2022; Zhang‐Yi et al. 2021; Zhang et al. 2023) and (5) dynamism (Babamohamadi et al. 2022; Kalkim, Sagkal Midilli, and Daghan 2018; Kang et al. 2021; Petersen and Schiltz 2020; Ross et al. 2022; Vogel and Schep‐Akkerman 2018). In summary, we propose that spiritual competence in nursing is a dynamic process that nurses must initiate by cultivating their own spiritual awareness. Once cultivated, this awareness can enable nurses to acquire spiritual well‐being for themselves as well as the knowledge, skills and sense of self‐efficacy that are critical in their provision of spiritual care. The way in which this process unfolds is unique for each nurse.

Spiritual awareness is required to initiate the dynamic process of spiritual competence. Spiritual awareness related to nursing typically evolves in the context of nurses' professional experiences (Ross et al. 2016; Sezer and Ozturk Eyimaya 2022; van der Vis‐Sietsma, de Man‐van Ginkel, and van Leeuwen 2019; Zhang et al. 2023). Spiritual awareness is a critical initiating factor because it enables nurses to understand, accept and respect their own spiritual beliefs, the spiritual beliefs of others and the differences among them. By appreciating these differences, nurses can begin to holistically assess patients' spiritual needs (Ahmadi et al. 2021; Baldacchino 2006; Damsma‐Bakker and van Leeuwen 2021; Guo et al. 2023). Researchers have found that spirituality‐related assignments in nursing‐education programs can improve students' spiritual awareness and help them view patients from a more holistic perspective (van der Vis‐Sietsma, de Man‐van Ginkel, and van Leeuwen 2019).

Nurses who possess spiritual well‐being experience satisfaction (Azarsa et al. 2015; Jafari and Fallahi‐Khoshknab 2021; Ross et al. 2016, 2018) are in a better position to recognise and meet the spiritual‐care needs of patients (Bangcola 2022; Özakar Akça, Gülnar, and Özveren 2022; Ross et al. 2014). Jafari and Fallahi‐Khoshknab (2021) found that nurses with higher levels of spiritual well‐being exhibit higher levels of spiritual competence. Similarly, Azarsa et al. (2015) found that nurses' spiritual well‐being bolsters their spiritual awareness and professional experience in ways that improve the nurses' ability to recognise patients' spiritual care needs.

Nurses acquire the ‘spiritual knowledge and skills’ required for spiritual competence through education and clinical training that address spirituality and spiritual care (Irmak and Sagkal Midilli 2021; Kang et al. 2021; Özakar Akça, Gülnar, and Özveren 2022; Petersen and Schiltz 2020; Zhang et al. 2023). When equipped with spiritual knowledge and skills, nurses can effectively harness their spiritual awareness and well‐being to provide spiritual care tailored to patients' spiritual needs (Babamohamadi et al. 2022; Heidari, Afzoon, and Heidari 2022; Hellman, Williams, and Hurley 2015; Kalkim, Sagkal Midilli, and Daghan 2018; Wang et al. 2022). Connors, Good, and Gollery (2017) found that innovative teaching strategies such as spiritual assessment and spiritual care simulation can improve nursing students' knowledge and skills necessary for listening to patients, assessing their spiritual needs and providing quality spiritual care.

Spiritual‐care self‐efficacy refers to nurses' belief that they are competent providers of spiritual care (Shamsi, Khoshnood, and Farokhzadian 2022; Southard et al. 2020; Wang et al. 2022; Zhang‐Yi et al. 2021). This belief can be thought of as confidence in providing spiritual care and has been found to be positively associated with trust between nurses and patients (Kalkim, Sagkal Midilli, and Daghan 2018; Petersen et al. 2017; Sezer and Ozturk Eyimaya 2022; Zhang et al. 2023). The more nurses believe in their ability to provide spiritual care, the more they practice it, which further improves their ability to provide spiritual care (Azarsa et al. 2015; Cheng et al. 2021; Guo et al. 2021; Heidari, Afzoon, and Heidari 2022). Nursing students who are more confident in their ability to provide spiritual care are better able to provide it and connect with patients (Ross et al. 2014).

Spiritual competence in nursing is a dynamic process even though only one of the reviewed studies supports this characterisation (Ross et al. 2022). Evidence of dynamism is demonstrated through the cumulative findings of all the studies reviewed for this analysis. Reviewed studies consistently report that nurses' spiritual competence evolves over time. It has neither a defined endpoint nor a universally applied path. Rather, the development and evolution of the attributes of spiritual competence in nursing follow the highly indeterminate fluctuations of nurses' professional careers and the contexts in which their careers play out.

### 3.4 Antecedents

Antecedents are events that occur before a concept and contribute to the occurrence of the concept (Tofthagen and Fagerstrøm 2010). We identified the following three antecedents of spiritual competence in nursing: (1) nurses' willingness to learn about spirituality (Guo et al. 2023; Kalkim, Sagkal Midilli, and Daghan 2018; Karaman and Sagkal Midilli 2022; Li et al. 2022; Sezer and Ozturk Eyimaya 2022); (2) nurses' engagement in spiritual education (Connors, Good, and Gollery 2017; Damsma‐Bakker and van Leeuwen 2021; Petersen et al. 2017; Petersen and Schiltz 2020; Ross et al. 2022; Shamsi, Khoshnood, and Farokhzadian 2022; Southard et al. 2020; van der Vis‐Sietsma, de Man‐van Ginkel, and van Leeuwen 2019; van Leeuwen et al. 2008) and (3) nurses' engagement in spiritual‐care training (Damsma‐Bakker and van Leeuwen 2021; Petersen et al. 2017; Shamsi, Khoshnood, and Farokhzadian 2022; van der Vis‐Sietsma, de Man‐van Ginkel, and van Leeuwen 2019). The three antecedents occur sequentially: willingness precedes and promotes engagement in spiritual education, which itself precedes and promotes engagement in spiritual‐care training.

Willingness to learn about spirituality precedes and promotes nurses' spiritual competence (Guo et al. 2023; Kalkim, Sagkal Midilli, and Daghan 2018). For instance, the more open a nurse is to receiving education and training related to spirituality and spiritual care, the more spiritually competent the nurse tends to be (Karaman and Sagkal Midilli 2022). The more enthusiasm or desire there is in a nurse's willingness to learn about spirituality and spiritual care, the better able the nurse will be to grasp relevant complex concepts (Li et al. 2022; Sezer and Ozturk Eyimaya 2022). Willingness to learn about spirituality may nurture positive attitudes toward spiritual care, contributing to nurses' spiritual competence (Guo et al. 2023; Karaman and Sagkal Midilli 2022).

Multiple studies have examined how spiritual education can shape spiritual competence in nursing (Petersen and Schiltz 2020; Ross et al. 2022; Shamsi, Khoshnood, and Farokhzadian 2022; Southard et al. 2020; van der Vis‐Sietsma, de Man‐van Ginkel, and van Leeuwen 2019; van Leeuwen et al. 2008). Spiritual education for nurses can strengthen their theoretical understanding of both spirituality (Petersen et al. 2017; Ross et al. 2022; Southard et al. 2020; van Leeuwen et al. 2008) and spiritual care (Petersen et al. 2017; Ross et al. 2022; Shamsi, Khoshnood, and Farokhzadian 2022; van der Vis‐Sietsma, de Man‐van Ginkel, and van Leeuwen 2019). Innovative teaching methods can be used to improve nursing students' spiritual competence. For instance, Connors, Good, and Gollery (2017) found that students' exposure to a spiritual education may significantly improve the confidence with which they view their spiritual‐care abilities. In another study, Damsma‐Bakker and van Leeuwen (2021) implemented an online spiritual‐care competency tool for nursing students that enhanced their awareness of the importance of spiritual care in nursing.

Spiritual‐care training, which nursing students or nurses receive in clinical settings after they have completed some form of spiritual education, focuses on the practical skills that these individuals will need if they are to provide high‐quality spiritual care (Petersen et al. 2017; Shamsi, Khoshnood, and Farokhzadian 2022). Nursing students and nurses who receive spiritual‐care training can move beyond any fear they might have regarding their ability to provide quality spiritual care (Damsma‐Bakker and van Leeuwen 2021; van der Vis‐Sietsma, de Man‐van Ginkel, and van Leeuwen 2019). Spiritual‐care training can help career nurses develop spiritual competency (Shamsi, Khoshnood, and Farokhzadian 2022). For example, a nursing‐education program that balances the theoretical component of spiritual learning with supervised training activities involving a real patient alerted nursing students to the benefits of spiritual care and improved their spiritual competence, which included the ability to communicate with patients about spiritual matters (van der Vis‐Sietsma, de Man‐van Ginkel, and van Leeuwen 2019).

### 3.5 Consequences

Consequences are events that can occur as a result or outcome of a concept (Tofthagen and Fagerstrøm 2010). The consequences of spiritual competence in nursing can be personal or professional. Personal consequences include enhanced well‐being, spiritual health, satisfaction and personal growth (Babamohamadi et al. 2022; Bangcola 2022; Chen et al. 2020; Damsma‐Bakker and van Leeuwen 2021; Guo et al. 2021, 2023; Li et al. 2022; Machul et al. 2022; Petersen and Schiltz 2020; van der Vis‐Sietsma, de Man‐van Ginkel, and van Leeuwen 2019), while professional consequences include an improved ability to provide holistic care, patient‐centred care and improved patient‐health outcomes (Abell, Garrett‐Wright, and Abell 2018; Abu‐Snieneh and Abdelaziz 2022; Adib‐Hajbaghery, Zehtabchi, and Fini 2017; Ahmadi et al. 2021; Baldacchino 2006; Chen et al. 2020; Cheng et al. 2021; Damsma‐Bakker and van Leeuwen 2021; Guo et al. 2021, 2023; Heidari, Afzoon, and Heidari 2022; Hsieh et al. 2020; Jafari and Fallahi‐Khoshknab 2021; Kang et al. 2021; Li et al. 2021; Machul et al. 2022; Petersen et al. 2017; Petersen and Schiltz 2020; Ross et al. 2016; Semerci et al. 2021; Southard et al. 2020; van der Vis‐Sietsma, de Man‐van Ginkel, and van Leeuwen 2019; van Leeuwen et al. 2008; Zhang‐Yi et al. 2021; Zhang et al. 2023).

From a personal perspective, acquiring spiritual competence enhances nurses' spiritual well‐being and confidence in providing spiritual care, two outcomes that reduce feelings of frustration that nurses might experience while providing spiritual care (Babamohamadi et al. 2022; Damsma‐Bakker and van Leeuwen 2021; Guo et al. 2021; Heidari, Afzoon, and Heidari 2022). Because spiritual well‐being in nurses can take the form of emotional peace or low work‐related stress, spiritual well‐being improves their ability to address patients' spiritual needs (Heidari, Afzoon, and Heidari 2022; Zhang‐Yi et al. 2021). Spiritually competent nurses are relatively satisfied with their performance of nursing duties, including spiritual nursing duties, and are in a good position to experience personal growth, to endure life stressors and to provide high‐quality nursing care (Bangcola 2022; Chen et al. 2020; Machul et al. 2022; van der Vis‐Sietsma, de Man‐van Ginkel, and van Leeuwen 2019).

Spiritually competent nurses are likely to provide holistic focused care by addressing patients' spiritual needs, in addition to their physical and psychosocial needs (Abell, Garrett‐Wright, and Abell 2018; Ahmadi et al. 2021; Baldacchino 2006; Chen et al. 2020; Jafari and Fallahi‐Khoshknab 2021). The spiritual‐focused care provided by spiritually competent nurses is likely to be tailored to patients' unique needs in all stages of illness, a patient‐centred approach that improves health outcomes (Baldacchino 2006; Damsma‐Bakker and van Leeuwen 2021; Guo et al. 2023; Machul et al. 2022; Ross et al. 2016). In general, spiritual care provided by spiritually competent nurses can improve patients' quality of life by decreasing their suffering, increasing their hope and fostering their acceptance of life circumstances (Abu‐Snieneh and Abdelaziz 2022; Adib‐Hajbaghery, Zehtabchi, and Fini 2017; Guo et al. 2021; Heidari, Afzoon, and Heidari 2022; Kang et al. 2021; Li et al. 2021; Petersen et al. 2017; Petersen and Schiltz 2020).

### 3.6 Proposed Conceptual Definition

Based on the above definitions and findings regarding spiritual competence in nursing, we define the concept as *a dynamic process that, once initiated by nurses' spiritual awareness, promotes their spiritual well‐being, their spiritual knowledge and skills, and their sense of spiritual‐care self‐efficacy*.

## 4 Discussion

Our concept analysis clarified attributes, antecedents and consequences of spiritual competence in nursing and supported the development of a clear definition of the concept that should strengthen research on the topic and actual practice in the field. We identified five attributes of spiritual competence in nursing: spiritual awareness, spiritual well‐being, spiritual knowledge and skills, spiritual‐care self‐efficacy and dynamism. Although only one of the reviewed studies explicitly included dynamism, findings from almost all of the reviewed studies implicitly indicate the dynamic evolution of spiritual competence.

In analysing the literature, we identified three antecedents of spiritual competence in nursing: nurses' willingness to learn about spirituality, nurses' engagement in spiritual education and nurses' engagement in spiritual‐care training. These antecedents may be influenced by nurses' spiritual, religious or philosophical beliefs. This may be more common in non‐religious contexts (i.e., countries without a state religion), where culture, values and philosophical beliefs may provide a foundation for enhancing spiritual competence for some nurses. To become spiritually competent, nurses should receive some form of education and training in the delivery of spiritually focused nursing care. However, mandating that nurses participate in competency development may be counterproductive, as willingness to receive an education and to participate in training are positively associated with the degree of spiritual competence. Thus, efforts should be made to provide spiritual education and training to nursing students and practicing nurses who are willing to engage in these development programs. The consequences of spiritual competence in nursing can be broken down into personal and professional consequences. Among the most frequently cited personal consequences are enhanced well‐being, satisfaction and personal growth, while the most important professional consequence is improved patient health outcomes. These consequences reflect the positive components of spirituality, such as acquiring meaning and purpose in life and promoting transcendence and connectedness with the self, community and the sacred.

Sociodemographic and professional factors associated with spiritual competence in nursing include age, gender, marital status, religion, educational level, nursing work‐related experience and frequency of spiritual practices (Adib‐Hajbaghery, Zehtabchi, and Fini 2017; Alshehry 2018; Babamohamadi et al. 2022; Chen et al. 2020; Guo et al. 2021; Hsieh et al. 2020). Participants in the selected studies covered a wide age range, and multiple studies suggest that nurses between 20 and 50 years of age are more likely than nurses over the age of 50 to be spiritually competent (Adib‐Hajbaghery, Zehtabchi, and Fini 2017; Guo et al. 2021; Hsieh et al. 2020; Irmak and Sagkal Midilli 2021; Kang et al. 2021). Although most participant‐based studies included primarily female participants, which precluded an examination of possible differences between males and females, one study found evidence that female nurses are significantly more spiritually competent than male nurses (Babamohamadi et al. 2022). Studies had inconsistent findings regarding marital status, with Guo et al. (2021) and Kang et al. (2021) proposing that married nurses are more spiritually competent than single nurses, while Babamohamadi et al. (2022) found that single nurses are more spiritually competent than married nurses. Although most participants in the relevant studies identified as religious, only two studies found that these participants were significantly more spiritually competent than participants who did not identify as religious (Hsieh et al. 2020; Machul et al. 2022). Regarding educational levels, those with higher educational levels are more spiritually competent. For example, Chen et al. (2020) and Guo et al. (2021) presented evidence that nurses with a bachelor's degree are more competent than those with an associate degree, while Kang et al. (2021) reported that nurses with a master's degree are more spiritually competent than those with a bachelor's or an associate degree. Nursing experience is also associated with spiritual competence: nursing experience has been positively and significantly associated with spiritual competence (Adib‐Hajbaghery, Zehtabchi, and Fini 2017; Guo et al. 2021; Hsieh et al. 2020; Kang et al. 2021). Experience with spiritual activities (e.g., reading books on the subject, meditating and practicing mindfulness) was found to enhance nurses' spiritual competence (Chen et al. 2020; Hsieh et al. 2020). While these findings provide preliminary evidence that sociodemographic and professional factors may affect nurses' spiritual competence, additional research is needed to better understand the unique contributions that these factors make to spiritual competence in nursing.

Findings from this analysis should be interpreted in the context of the specific nursing role. Of all the studies, 69% recruited practicing nurses (i.e., nurses who were working directly with patients); the remaining 31% recruited nursing students. Although the aim of this study was to provide a comprehensive understanding of the concept of spiritual competence in nursing, we may have overlooked factors specific to being a nurse (e.g., salary level) or being a nursing student (e.g., grade point average) that might shape development of spiritual competence. In addition, most of the selected studies enrolled practicing nurses from multiple departments; only a few studies enrolled nurses from specific specialty departments, such as hospice (Kang et al. 2021); oncology (Damsma‐Bakker and van Leeuwen 2021); intensive care (Abu‐Snieneh and Abdelaziz 2022) and psychiatry (Irmak and Sagkal Midilli 2021). Thus, further research is needed to explore whether department‐specific factors influence the spiritual competence of nurses caring for specific populations.

Ability and opportunity to provide spiritual care are essential to the process of developing spiritual competence in nursing. Thus, impediments to these factors can undermine nurses' spiritual competence. In the studies analysed, the impediments that were cited include onerous workloads, fear of overstepping patients' values, lack of confidence in providing spiritual care and insufficient knowledge about spirituality and the provision of spiritual care. In education and training programs for nurses, an emphasis on spirituality may help these individuals overcome some of the above impediments.

Attention should be paid to the conceptual models that underpin educational and training programs devoted to nurses' spiritual competence, as they can affect the development, implementation and outcomes of this competence. Our analysis of the selected studies led us to identify three conceptual models of spiritual competence that could facilitate the development of such programs. The Spiritual Competence Model for Psychiatric care (Campinha‐Bacote 1995) includes three critical attributes of spiritual competence in nursing: spiritual awareness and spiritual knowledge and spiritual skills. Although the model was initially developed for psychiatric nurses, its use in general nursing gives every indication of being valid. The model could be used to help develop spirituality‐oriented educational and training programs, including those related to the development of spiritual assessments and spiritual care in nursing. Another model—the Spiritual Care Competency Framework—consists of seven domains: (1) knowledge in spiritual care; (2) self‐awareness in spiritual care; (3) interpersonal relationships and communication; (4) ethical and legal issues in spiritual care; (5) assessment and implementation of spiritual care; (6) quality assurance in spiritual care and (7) informatics in spiritual care (Attard, Ross, and Weeks 2019). This framework can facilitate the design and delivery of educational and training programs targeting spiritual care competency. Finally, Cao et al. (2020) developed the Spiritual Care Competence Conceptual Framework to help educators develop a program for teaching spiritual care to practicing nurses in China: the framework focuses on (1) nurses' awareness, understanding and application of spirituality; (2) the intrapersonal, interpersonal and transpersonal levels of spirituality in nurses and (3) the worldview, connectedness and transcendence levels of spirituality in nurses. This framework can guide the design of clinical training programs for the spiritual‐care competence of nurses. Further research on these models can determine the extent to which they can effectively guide the education and training of diverse populations of nursing students and practicing nurses in the realm of spiritual competence.

### 4.1 Limitations

The studies selected for this concept analysis each had limitations, which imposed important limitations on our findings. Most of the selected studies used the SCCS to operationalise care‐focused spiritual competence. However, because the SCCS measures skills, it is unlikely to measure all five attributes of spiritual competence identified in our analysis. As recommended by van Leeuwen et al. (2009), researchers seeking to measure our broader range of attributes should consider combining the SCCS with other scales (particularly those that measure spiritual awareness and spiritual attitudes). In addition, most studies measured only the ‘levels’ of spiritual competence, a pattern that left unaddressed other aspects of spiritual competence, especially its consequences.

The cultural and religious contexts of the selected studies could also have affected our analysis. Most of these studies were conducted in countries where (1) many people consider religion an essential element of one's identity; (2) many people treat religion and spirituality interchangeably and (3) the majority of participants identified as religious. Studies conducted in these countries found evidence that religion significantly affects nurses' spiritual competence, but generally left unanswered the question of how non‐religious nurses and nursing students perceive and respond to issues involving spiritual competence. Another limitation of our study is that all of our selected studies were in English, and this fact may have led us to exclude important research published in other languages. Finally, we analysed only those studies addressing the nursing discipline. This decision limits the generalizability of our findings, as practitioners in other disciplines may hold views of spiritual competence quite different from those of nurses.

### 4.2 Implications for Nursing

Spiritual competence in nursing is essential to nurses' personal and professional development and to providing holistic patient‐centred care. Integrating spirituality into nursing education may serve as a foundation for clinical practice and research, where nurses demonstrate their spiritual competence. Thus, we highlight the significant implications of spiritual competence for nursing education and training, nursing assessment and management and nursing research.

#### 4.2.1 Nursing Education and Training

As mentioned above, spiritual care is a required nursing competency in nursing education (American Association of Colleges of Nursing 2021) and in providing holistic patient‐centred care (American Holistic Nurses Association 2016; Hospice and Palliative Nurses Association 2022). However, most US undergraduate nursing programs and textbooks do not address spirituality as part of formal nursing education. To address this, nursing courses and curricula should include formal instruction on fundamental aspects of spirituality (e.g., spiritual awareness and spiritual care in nursing) for all stages of spiritual development across the lifespan. Integrating spirituality in nursing educational programs will allow nursing students to develop their spiritual awareness, making them better equipped to provide holistic patient‐centred care.

There is a need to integrate spirituality into clinical training programs to enhance nurses' spiritual competency post‐graduation. This may be most effective when training and education on spirituality are implemented in care settings or contexts that require spiritual care, such as long‐term care facilities (Chen et al. 2020), critical care units (Abu‐Snieneh and Abdelaziz 2022; Azarsa et al. 2015), psychiatry units (Irmak and Sagkal Midilli 2021) and units providing hospice or end‐of‐life care (Kang et al. 2021). The formal inclusion of spirituality into nursing instruction and clinical training programs can be guided by one of the conceptual models identified earlier, such as the Spiritual Care Competency Framework (Attard, Ross, and Weeks 2019). Holistic nursing clinical training programs that promote nurses' spiritual competence may assist nurses in navigating patients' needs and care.

#### 4.2.2 Nursing Assessment and Management

A comprehensive assessment of patients' spiritual needs is essential to providing spiritual care. Integrating comprehensive standard spiritual assessments into clinical nursing practice will facilitate nurses' provision of spiritual care and equip nurses with the skills to collaborate with and refer patients to chaplains and other religious and spiritual professionals as needed (Campbell, Robison, and Godsey 2023). Yet, institutional guidance pertaining to spiritual assessment for nurses is often lacking (Balboni et al. 2022). The lack of institutional guidance with the current implementation of spiritual care provides opportunities for nurse leaders to define who should assess patients' spiritual needs, how often assessment should occur and what types of care or interventions can be offered to meet identified spiritual needs. Nurse leaders and policymakers should ensure that spiritual care policies reflect evidence‐based recommendations.

#### 4.2.3 Nursing Research

The findings of this concept analysis highlighted limitations in the analysed studies that serve as areas for future research. For example, it was not clear whether nurses' sociodemographic characteristics significantly influence their spiritual competence, particularly within nurses' cultural and religious contexts. Thus, further targeted research is needed to explore the potential role of nurses' sociodemographic characteristics in more diverse samples (e.g., for nurses who do not identify as religious). Additionally, further research is needed to explore nurses' spiritual competence in specialty areas (e.g., oncology) to understand patterns of patients' spiritual needs in these units. Lastly, further research is needed to examine the feasibility of spiritual assessment tools before their implementation into clinical practice.

In conclusion, findings from our concept analysis help clarify the concept and related practices of spiritual competence in nursing. We have shown that spiritual competence is a dynamic process initiated by spiritual awareness and promotive of ever‐evolving spiritual well‐being, spiritual knowledge and skills, and spiritual‐care self‐efficacy in the lives of nursing students and practicing nurses. Our concept analysis provides a foundation for future research aimed at helping nurses develop spiritual competence and provide quality spiritual care.

## Author Contributions

This manuscript was written by N.S.A. with a critical review from A.L.E. and K.E.M. N.S.A. and A.L.E. contributed to the study's conception and design. Data collection and analysis were performed by K.E.M. and N.S.A. All authors commented on previous revisions and provided approval for the final version for publication.

## Conflicts of Interest

The authors declare no conflicts of interest.

## Data Availability

Data sharing is not applicable to this article as no new data were created or analyzed in this study.
